# Cost-comparison analysis of a multiplatform tumour profiling service to guide advanced cancer treatment

**DOI:** 10.1186/s12962-019-0191-6

**Published:** 2019-10-21

**Authors:** Gilbert Spizzo, Uwe Siebert, Guenther Gastl, Andreas Voss, Klaus Schuster, Robert Leonard, Andreas Seeber

**Affiliations:** 1 Department of Internal Medicine, Oncologic Day Hospital, Bressanone Hospital (SABES-ASDAA), Bressanone-Brixen, Italy; 2 Department of Haematology and Oncology, Innsbruck Medical University, Innrain 66, 6020 Innsbruck, Austria; 3 Institute of Public Health, Medical Decision Making and HTA, Hall in Tirol, Austria; 4 Caris Life Sciences, Basel, Switzerland; 5 Imperial College, London, UK; 6 Laboratory for Oncogenomics, Tyrolean Cancer Research Institute, Innsbruck, Austria

**Keywords:** CARIS multiplatform, Molecular profiling, Cost-effectiveness, Cancer treatment

## Abstract

**Background:**

Tumor profiling is increasingly used in advanced cancer patients to define treatment options, especially in refractory cases where no standard treatment is available. Caris Molecular Intelligence (CMI) is a multiplatform tumor profiling service that is comprehensive of next-generation sequencing (NGS) of DNA and RNA, immunohistochemistry (IHC) and in situ hybridisation (FISH). The aim of this study is to compare costs of CMI-guided treatment with prior or planned treatment options in correlation with outcome results.

**Methods:**

Retrospective data from five clinical trials were collected to define the treatment decision prior to the receipt of the CMI report (n = 137 patients). A systematic review of treatment data from 11 clinical studies of CMI (n = 385 patients) allowed a comparison of planned vs actual (n = 137) and prior vs actual (n = 229) treatment costs.

**Results:**

Treatment plan was changed in 88% of CMI-profiled cases. The actual CMI guided treatment cost per cycle was £995 in 385 treated patients. Planned treatment costs were comparable to actual treatment costs (£979 vs £945; p = 0.7123) and prior treatment costs were not significantly different to profiling-guided treatments (£892 vs £850; p = 0.631).

**Conclusions:**

Caris Molecular Intelligence guided treatment cost per cycle was in the range of prior or planned treatment cost/cycle. Due to beneficial overall survival the additional cost of performing CMI’s multiplatform testing to the treatment costs seems to be cost-effective.

## Background

Tumour profiling in oncology involves the use of high throughput technologies such as next-generation sequencing (NGS) of DNA and/or RNA, immunohistochemistry (IHC) and in situ hybridization (FISH) beside others. These techniques are being used as precision tools to predict which treatments may be beneficial for an individual patient or potentially lack benefit. Nowadays, there are different molecular profiling services available to oncologists and they differ greatly in the technologies used, reported results and the costs associated with subsequent treatment options [1].

Predictive associations for conventional chemotherapeutic and anti-hormonal agents are mostly based on the alterations in protein expression as determined by IHC. The identification of “druggable” genetic alterations by NGS-only leads typically to the recommendation of relatively recently introduced targeted therapy drugs, which are usually much more expensive [2].

The integration of profiling into clinical routine is hindered mainly by the lack of insurance coverage (public reimbursement) and the perceived high costs of the profiling test itself. Various health economic models have been developed for the introduction of a new drug or a diagnostic test into a healthcare system. However, the introduction of tumour profiling creates some difficulties as it is offered for a wide range of solid tumours and the consequent variable costs of the drugs that are recommended. Moreover, there are different costs depending on the type of test used for profiling. Recent data showing the health-economic impact of molecular profiling has focused on incremental increases in progression-free survival (PFS), total costs and cost per week of survival associated with profiling-guided therapies [3].

Caris Molecular Intelligence (CMI) is a multiplatform tumour profiling service [4] that is used for guiding treatment options in patients with advanced cancer. Caris Life Sciences has been establishing a post-marketing registry with the aim to perform multicentre prospective observational studies to support an ongoing clinical outcome database. In one analysis of the Caris registry data, the median overall survival (OS) in the patients without selection based on matched and unmatched treatment recommendation is 931 days. The OS in patients who received matched therapy is 1069 days indicating that CMI can extend overall survival by 138 days (0.378 years). In addition, CMI can identify the treatments associated with lack of benefit and thus reduce spending on ineffective drugs and adverse effects. The median OS in the unmatched cohort is 686 days, 245 (0.67 year) less than the median OS in the unselected population [5].

As such, the Caris registry is unique, as it gives for the first time the opportunity to assess the benefits of broad molecular profiling in relation to the costs of the test itself and treatment costs.

The aim of this study is to compare the costs of CMI-guided treatments and treatments of physician’s choice with actual or planned treatments. This should allow to indicate potential cost-effectiveness of the multiplatform tumour profiling service of Caris Molecular Intelligence.

## Patients and methods

The treatments administered following profiling were collected from 11 studies using Caris Molecular Intelligence [6–11]. In five of these studies, the treatment that was planned in the absence of profiling (i.e. the treatment of physician’s choice) was recorded. The treatment decision was considered changed if at least one component of the treatment regimen was different to the planned treatment. Taken together, data from 385 CMI profiled patients were collected. The prior line of treatment was documented in 229 patients within this cohort. The planned treatment was recorded in 137 patients.

The average cost per treatment cycle was calculated from the British National Formulary BNF (version 70 dated March 2016) and based on a treatment cycle of 21 days for all oral and systemic drugs. List price for CMI was used in the calculation of cost of treatment and testing per PFS gain. The list price for the United Kingdom was used (£5000). Cost of treatment per PFS week has been described as an index for assessing cost of care in precision medicine [3]. Prior PFS is assumed to be 90 days (~ 3 months) [12]. Subsequent unmatched PFS would be expected to be approximately one-third shorter and has been reported to be 49 days in a contemporary cohort [13]. Progression-free survival of CMI-guided treatment is postulated to be 120 days (~ 4 months) [14]. Finally, all patients would theoretically receive 4.2 cycles of treatment. Decision impact was assessed, comparing actual, planned and CMI-guided treatments. Statistical analysis (unpaired t-test) was performed using GraphPad™.

## Results

In 137 patients profiled with CMI, the treatment decision was changed in 120 cases (88%) and remained unchanged in 17 cases (12%). The patients’ characteristics are summarized in Table 1. The majority of CMI-guided treatments administered to the cohort of 385 patients consisted of chemotherapy alone or in combination, which is similar to the treatments administered previously (72% of 229 patients) and to planned treatments (66% of 137 patients).

**Table 1:** Patients characteristics

All cases	N = 137
Mean age (range)	59 (19–81)
Sex (m/f)	N = 61/76
Tumor type	
Gastrointestinal	N = 71
Breast cancer	N = 38
Other gynecologic cancer	N = 7
Lung cancer	N = 6
Others	N = 15

**Fig. 1:**
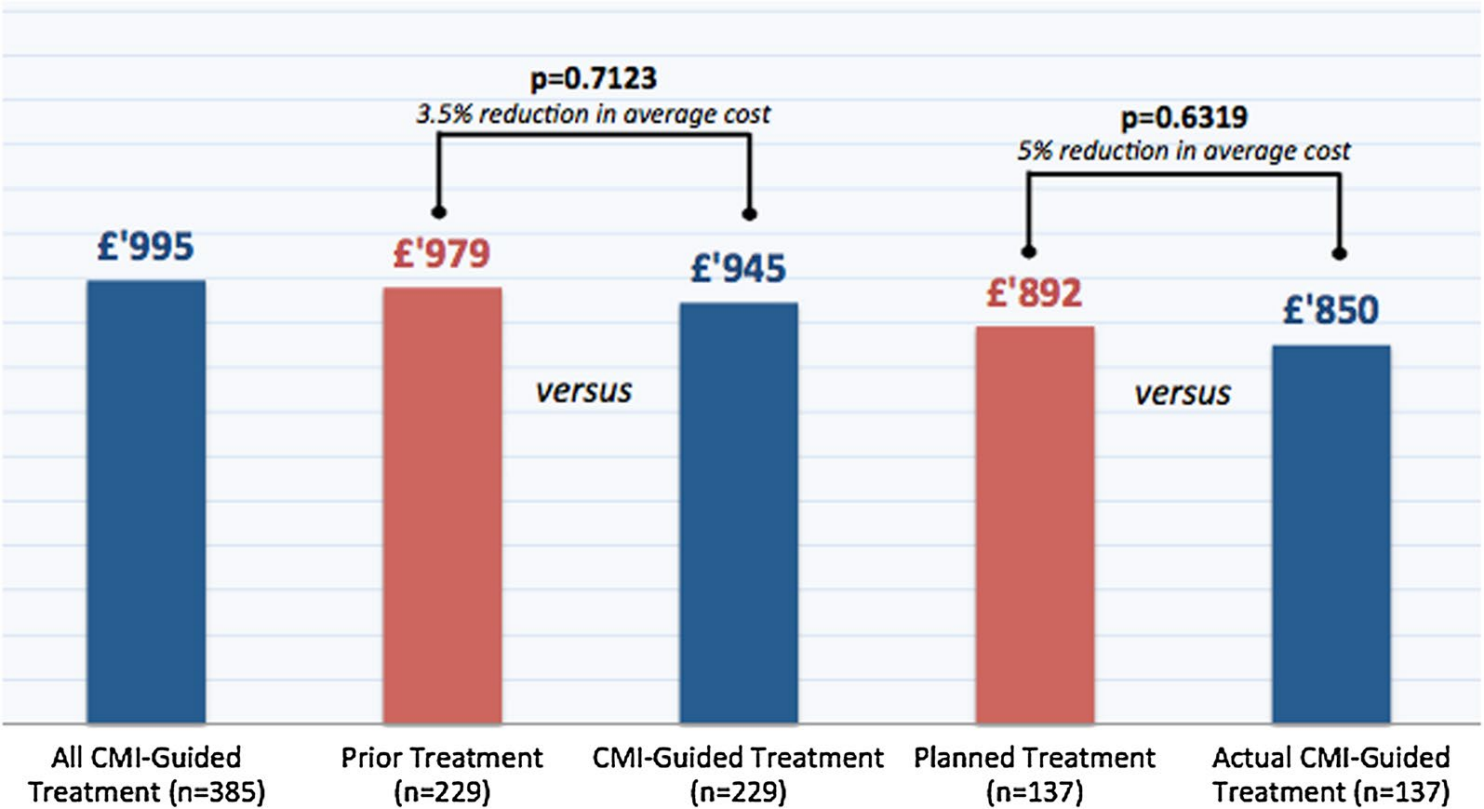
Comparison of CMI-guided treatment costs per cycle per patient to prior and planned treatments

The average treatment cost per patient per cycle of all CMI-guided therapies (n = 385) was £995 (range £3–£4446). The average treatment cost per patient per cycle in the prior line of treatment (n = 229) was £979 (range £44–£5651), compared to £945 in the same patients treated according CMI (range £3–£4.446, p = 0.7123). Although this difference was statistically not significant, there was a 3.5% reduction in average costs. The average treatment cost per patient per cycle for planned treatment (n = 137) was £892 (range £37–£5651, p = 0.613). Although this difference was not a statistically significant, an average cost reduction of 5% was observed (Fig. 1).

The planned treatment shows an average cost of £538 per week of PFS gained, whereas the average cost of the prior line of treatment was £321 per week of PFS gained and is 40% lower than planned costs. The costs of CMI testing and CMI-guided treatment amounted to an average of £500 per week of PFS gained, 7% lower than planned treatment costs.

In the CARIS registry data, the median OS in patients without CMI-guided treatment is 931 days. The OS in patients who received matched therapy is 1069 days indicating that CMI can potentially extend OS by 138 days (0.378 years). The median OS in the unmatched cohort is 686 days, 245 (0.67 year) less than the median OS in the unselected population. Globally, CMI-guided treatment was administered in 77% of profiled patients.

**Fig. 2:**
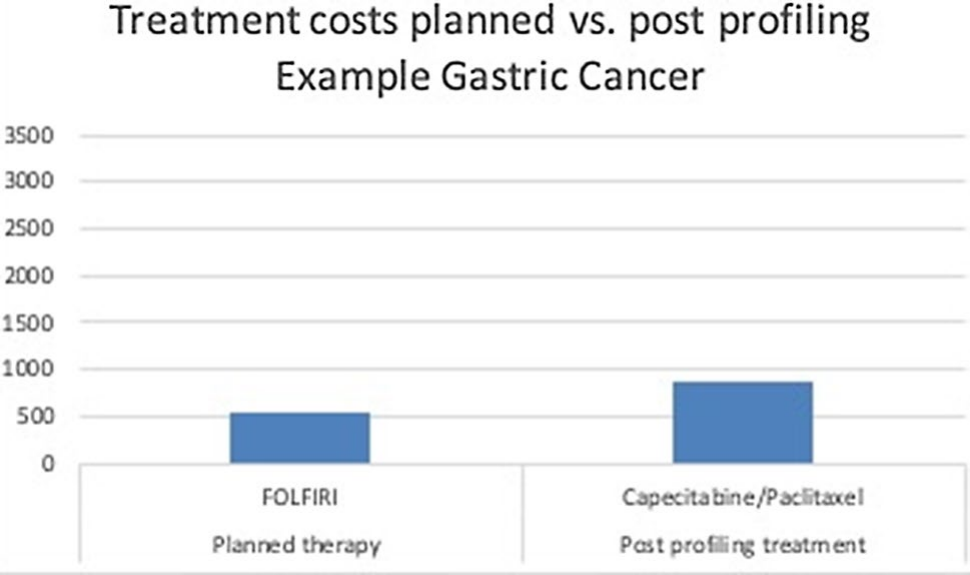
Example of planned vs post-profiling treatment costs of a patients with gastric cancer. Treatment with FOLFIRI is calculated with £550.9 and the combination of capecitabine with paclitaxel £876.75

**Fig. 3:**
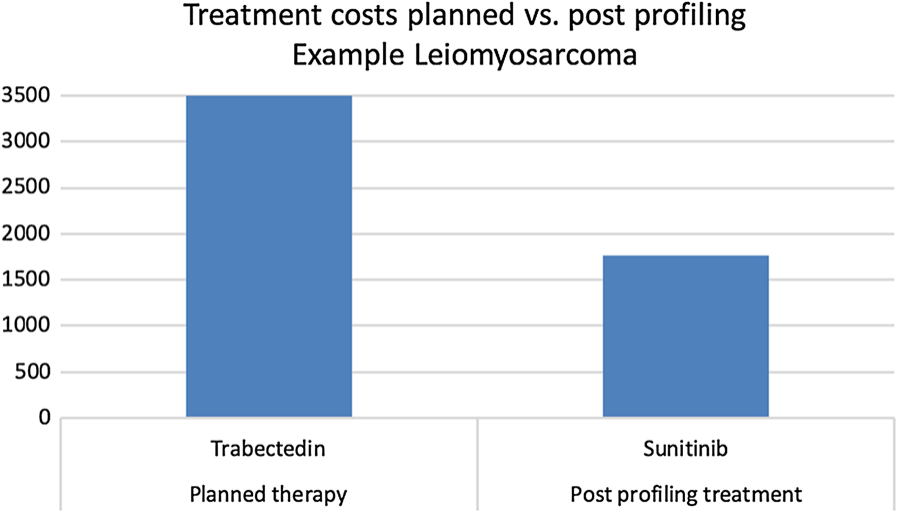
Example of planned vs post-profiling treatment costs of a patients with leiomyosarcoma. Trabectedin is calculated with £3544.77 and sunitinib 1765.58

Finally, two examples of a planned and actual guided-treatment plan in terms of units of medicines with associated unit prices are illustrated in Figs. 2  and 3.

## Discussion

Caris Molecular Intelligence (CMI) is a test that is used to help guide management of advanced cancer patients fit for further treatment (based on clinical ECOG-status, estimated life-expectancy and quality of life). Upon testing of formalin-fixed paraffin-embedded (FFPE) cancer tissue, Caris provides a report that describes biomarker results leading potentially to cancer treatments associated with increased or decreased likelihood of benefit, based on molecular and phenotypic characteristics of the tumour, taking the primary tumor site into consideration as well. Until now, no published evidence is available on the health economic impact of CMI. One may argue that CMI has potential for resource-allocation optimisation, since the test would prevent cancer patients from initiating treatments that offer little or no benefit. Other specialists may discuss, that the CMI test itself is rather expensive and that these costs finally have to be added to the overall economic burden of cancer treatment and that the test does not finally change the management of cancer patients.

However, the data of the present study shows that patients that have had a CMI test have a high probability to have their treatment plans changed (88%) as compared to the planned treatment before receiving the CMI report. This is a much higher percentage as observed with pure DNA-NGS only tests, where adapted, targeted treatment is performed in only 32% of tested individuals [15]. One of the major reasons for this high clinical utility is probably that CMI includes various technologies in its approach and not only NGS as profiling technique. The impact on treatment choice is directly dependent on the panel of biomarkers tested, the frequency of those biomarkers in the population and the level of evidence presented to the oncologist in support of a change in treatment decision. DNA next-generation sequencing only is mainly focused on targeted therapies, whereas CMI provides the most comprehensive information, including chemotherapies, endocrine treatments, immunotherapies as well as targeted therapies, analysing DNA, RNA and proteins with multiple technologies and continuous updates of the panel, reflecting latest evidence and scientific results. Hence, the clinical benefit of CMI test is probably the crucial variable that renders this test cost-effective.

Indeed, we could show that CMI-guided treatment is in line and within the range with previously planned treatments. To enable price comparison (cost per treatment cycle), all the drug regimens were based on 21 days cycles, which could represent a limitation of this cost comparison, because the effect of guided-therapy on duration of therapy is unknown. As all regimens were treated the same in all groups (actual, planned, CMI-guided treatments), the influence on the accuracy of the results should be minimal.

One major concern of this analysis is the great diversity of cancer patients included in studies on molecular profiling. Patients usually do not have the same stage or clinical presentation and this may hamper a proper comparison. Moreover, data regarding CMI-guided treatment in site-specific cancers are missing, which makes it difficult to compare it with approved drugs for single cancers. Furthermore, no data coming from randomized trials are available. Such data would be necessary to exclude a confounding bias such as survival outcome (observational vs prospective design).

Additionally, traditional cost-effectiveness analyses cannot be applied easily due to the fact, that tumor profiling tests report biomarkers. This information about biomarkers can then potentially influence the physician’s choice for treatment strategy but this information is only one factor driving the choice for treatment. Therefore, multi-dimensional rationales about the scope of tumor profiling, biomarker driven treatment options, cost-effectiveness on one hand and outcome results and quality of life-data on the other hand have to be implemented.

## Conclusions

Caris Molecular Intelligence-tumor profiling and its derived implications upon treatment strategies are not changing treatment costs/cycle significantly. Taken together, the additional cost of performing CMI’s multiplatform testing is may be cost-effective due to high clinical utility and additional potential overall survival benefit associated with the test. Further randomized studies for evaluating the cost-effectiveness of tumor profiling tests are needed to broaden access to such tests and enable discussions about reimbursement.

## Data Availability

The datasets used and/or analysed during the current study are available from the corresponding author on reasonable request.

## Abbreviations

CMI: Caris Molecular Intelligence
NGS: next generation sequencing
IHC: immunohistochemistry
FISH: fluorescence in-situ hybridization
OS: overall survival
PFS: progression free survival
BNF: British National Formulary
FFPE: formalin-fixed paraffin-embedded
